# The relationship between level of androgenic hormones and coronary artery disease in men

**Published:** 2007

**Authors:** Gholamreza Davoodi, Alireza Amirezadegan, Mohammad Ali Borumand, Maria Raissi Dehkordi, Ali Kazemisaeid, Ahmad Yaminisharif

**Affiliations:** Tehran Heart Centre, University of Tehran/Medical Sciences, Teheran, Iran; Tehran Heart Centre, University of Tehran/Medical Sciences, Teheran, Iran; Tehran Heart Centre, University of Tehran/Medical Sciences, Teheran, Iran; Tehran Heart Centre, University of Tehran/Medical Sciences, Teheran, Iran; Tehran Heart Centre, University of Tehran/Medical Sciences, Teheran, Iran; Tehran Heart Centre, University of Tehran/Medical Sciences, Teheran, Iran

## Abstract

**Background:**

Previous studies have shown controversial results on the role of androgens in coronary artery disease (CAD). We performed this study to assess the relationship between androgen levels and selective coronary angiography (SCA) findings.

**Methods:**

This study was conducted on 502 consecutive men who underwent SCA with different indications in our centre. Medical history and blood samples were taken from all subjects prior to angiography. F ree testosterone (FREET) was measured with enzyme-linked immunosorbent assay, and total testosterone (TES) plus dehydroepiandrosterone sulfate (DHEA) were checked with radio-immunoassay. Total cholesterol, high- and low-density lipoprotein cholesterol (HDL and L DL), triglycerides, lipoprotein (a) [Lp(a)] and C-reactive protein (CRP) were also tested in all patients. Angiographic results were reported by two cardiologists and checked for intra- and inter-observer reliability, then interpreted as Gensini score, and on the basis of the number of segments involved. The relationships were assessed with the chi-square test, independent sample *t*-test, one-way analysis of variances, Pearson’s correlation, and univariate and multivariate logistic regression tests.

**Results:**

Eighty-three (16.5%) of the subjects had single-vessel disease, 108 (21.5%) had two-vesssel, 197 (39.2%) had three-vessel disease, and 114 (22.7%) had normal angiograms or minimal lesions. F REET, TES and DHEA in patients with significant CAD vs normal individuals were 6.69 ± 3.20 pg/ml, 16.60 ± 6.66 nm/l and 113.38 ± 72.9 μg/dl vs 7.12 ± 3.58 pg/ml, 15.82 ± 7.26 nm/l and 109.03 ± 68.19 μg/dl, respectively (*p* > 0.1). There was no correlation between the Gensini score or the number of involved segments and androgen levels. Triglyceride, total cholesterol, L DL and HDL cholesterol levels also had no correlation with androgenic hormones. However, F REET showed a negative correlation with Lp(a) and CRP (*p* = 0.01, *r* = −0.12; *p* = 0.03, *p* = −0.096, respectively). Moreover, the level of DHEA was lower in diabetics (94.5 ± 59.19 μg/dl vs 117.97 ± 74.54 μg/dl, *p* = 0.004).

**Conclusions:**

There was no significant correlation between FREET, TES, DHEA and the presence or severity of CAD. Also, no correlation was found between androgen levels and triglyceride, total cholesterol, L DL and HDL cholesterol levels.

## Summary

Cardiovascular diseases are considered major health issues in many countries. Despite wide variations in absolute rates, global statistics show that men are 2.2 times (range 1.2–4.5) as likely as women to develop cardiovascular disease in many populations.^1^ This has largely been attributed to the established cardioprotective role of endogenous oestrogens.^2^-^6^ Likewise, testosterone has been proposed to play a protective role for the cardiovascular system in men. *In vitro* studies have demonstrated that testosterone may exert anti-atherogenic effects through vasorelaxation,^7^ with direct effects on the potassium channels of vascular smooth muscle cells,^8^ stimulation of nitric oxide release,^9^ and by a calcium antagonistic action.^10^ Testosterone has also been shown to have immuno-modulating effects with suppression of pro-inflammatory cytokines, which may have a role in the pathogenesis of coronary artery disease (CAD).^11^ Moreover, some studies have shown serum testosterone levels to have a direct relationship with plasma high-density lipoprotein (HDL) cholesterol, and an inverse relationship with low-density lipoprotein (LDL) cholesterol, total cholesterol, triglycerides, plasminogen activator inhibitor-1 activity, and fibrinogen.^12^

However, despite the established role of testosterone on vascular reactivity and the CAD risk profile, its effect on CAD has not been fully identified. There are contradictory results in this field, for example, some studies have shown that testosterone has a neutral or protective effect on the cardiovascular system.^12^ On the other hand, use of anabolic steroids has been shown to be responsible for sudden cardiac death, myocardial infarction and hypertension.^13^ Besides, some authors have proposed that myocardial infarction and chronic illnesses including CAD may lower androgen hormone levels,^14^ suggesting that low androgen levels may be a result rather than a cause of coronary artery disease.

In the current study, we aimed to determine the possible association between androgen levels and the presence or severity of CAD in the male Iranian population. As a secondary aim, the relationship of serum androgens with serum lipids and C-reactive protein (CRP) was investigated. Moreover, a subgroup study on the level of these hormones in diabetics was conducted.

## Patients

A total of 502 men were selected out of 719 consecutive male patients who underwent selective coronary angiography (SCA) in our centre between May and August 2005. Indications for coronary angiography were one or more of the following: typical chest pain, positive exertion stress test, positive myocardial perfusion scan, and history of myocardial infarction. Patients with chronic renal failure requiring dialysis, or those who were taking any medications known to affect sex hormone levels, for example, anti-androgen treatment for prostatic carcinoma or androgen preparations were excluded because of their effect on androgen levels. Moreover, patients with fever, major trauma or infections, and myocardial infarction within the preceding 14 days were excluded from the study because of the simultaneous measurement of CRP and the probable influence of these conditions on CRP levels. Informed, written consent was obtained from all participants. This study was approved by the Tehran Heart Centre ethics committee.

## Methods

Baseline data including age and history of risk factors for CAD were recorded. Height and weight were measured and body mass index was calculated by the formula: weight (kg)/height (m^2^). Early morning fasting blood samples (08:30–09:30) were taken immediately prior to coronary angiography, because the level of these hormones is at its peak in the early hours.

Patients were assessed for triglyceride, total cholesterol, LDL and HDL cholesterol levels, serum free testosterone (FREET), total testosterone (TES), dehydroepiandrosterone sulfate (DHEA), Lp(a), and CRP. Free testosterone was measured with enzyme-linked immunosorbent assay (ELISA) and total testosterone plus dehydroepiandrosterone sulfate were measured with radio-immunoassay (RIA). Total cholesterol and triglycerides were assessed with the enzymatic method. HDL cholesterol and LDL cholesterol were assayed using the direct method and Friedwald’s formula, respectively. Lp(a) was measured with enzyme-linked immunosorbent assay and CRP with immuno-turbidometry. ELISA kits were IBL for free testosterone and Biopool for Lp(a). RIA tests were performed with the Immunotech kit (France).

Two cardiologists reported the results of the SCA and the lesions were classified according to the clinical vessel score^15^ and Gensini score.^16^ The clinical vessel score, based on a scale of 0–3, was the number of vessels with a luminal diameter reduction of greater than 50%. So, clinically significant atherosclerosis was defined as one or more stenoses in one or more coronary arteries.

The Gensini score was computed by assigning a severity score to each coronary stenosis according to the degree of luminal narrowing and its geographic importance. Reduction in the lumen diameter, and the roentgenographic appearance of concentric lesions and eccentric plaques were evaluated (reductions of 25, 50, 75, 90, 99% and complete occlusion were given Gensini scores of 1, 2, 4, 8, 16 and 32, respectively). Each principal vascular segment was assigned a multiplier in accordance with the functional significance of the myocardial area supplied by that segment: the left main coronary artery, × 5; the proximal segment of the left anterior descending coronary artery (LAD), × 2.5; the proximal segment of the circumflex artery, × 2.5; the mid segment of the LAD, × 1.5; the right coronary artery, the distal segment of the LAD, the posterolateral artery, and the obtuse marginal artery, × 1; and others, × 0.5.

The angiography films were re-reported by the cardiologists, which yielded an intra- and inter-observer reliability of more than 95%.

## Statistics

Data were analysed using SPSS software version 13 and SAS software version 9.1. Categorical variables were analysed with chi-square and Fisher’s exact tests. The Student’s *t*-test was used for comparison of continuous variables. One-way analysis of variances (ANOVA) was used for comparison of hormone levels between patients with single-, two-, and three-vessel disease. Pearson’s correlation tests were used to find any correlation between particular continuous variables. A univariate logistic regression test was used for calculation of odds ratios and confidence intervals. Finally, presuming CAD as a dependent variable, we analysed the possible role of androgenic hormones on CAD after adjustment for age, hyperlipidaemia, diabetes mellitus, smoking, and levels of Lp(a), total cholesterol and LDL in a multivariable logistic regression model.

## Results

From 502 patients who were enrolled in this study, 83 (16.5%) had single-vessel disease, 108 (21.5%) had two-, and 197 (39.2%) had three-vessel disease. Of the total population, 114 (22.7%) had a normal angiogram or minimal lesions. In addition, 27 (5.3%) of all patients with CAD had left main stem lesions. Table 1 represents the baseline characteristics in the two groups with and without significant CAD. As expected, patients with CAD were older and had a higher frequency of established risk factors. Mean ejection fraction was lower in this group. BMI was not significantly different in these two groups.

**Table 1:** Baseline Characteristics In Patients With CAD And Normal Coronary Arteries

	*CAD*	*NCA*	*p*	*OR*	*95% CI*
Age (years)	57.0 ± 10.5*	52.9 ± 11.4	< 0.001	1.04	1.02−1.06
Diabetes mellitus	88 (23.1%)	12 (11%)	0.006	2.43	1.27−4.63
Hypertension	121 (31.8%)	20 (18.2%)	0.006	2.09	1.23−3.56
Hyperlipidaemia	147 (38.6%)	20 (18.3%)	< 0.001	2.79	1.65−4.73
Current smoking	125 (32.8%)	24 (21.8%)	0.027	1.75	1.61−2.88
Ejection fraction (%)	50 ± 11	56 ± 10	< 0.001	0.95	0.93−0.97
BMI (kg/m^2^)	28.05 ± 4.37	28.75 ± 4.75	0.14	−	−

*Mean ± standard deviation; CAD 5 patients with coronary artery disease; NCA 5 patients with normal coronary arteries.

Table 2 represents the concentration of androgenic hormones, CRP and lipids in both groups. As shown in Table 2, in men with CAD, the concentration of androgens was similar to those without significant CAD, although the concentrations of total cholesterol, LDL cholesterol and Lp(a) were significantly higher in patients with CAD.

**Table 2:** Hormone Profile And Other Laboratory Tests In Study Populations

	*CAD*	*NCA*	*p*	*OR*	*95% CI*
Free testosterone (pg/ml)	6.69 ± 3.20*	7.12 ± 3.58	0.22	−	−
Total testosterone (nm/l)	16.60 ± 6.66	15.82 ± 7.26	0.28	−	−
DHEA (μg/dl)	113.38 ± 72.9	109.03 ± 68.19	0.57	−	−
Lp(a) (mg/dl)	19.95 ± 14.14	16.34 ± 11.43	0.013	1.02	1.002−1.04
CRP (mg/dl)	11.52 ± 12.90	9.57 ± 6.65	0.12	−	−
Total cholesterol (mg/dl)	203.00 ± 46.00	190.35 ± 49.87	0.012	1.006	1.001−1.01
LDL cholesterol (mg/dl)	129.43 ± 38.75	116.42 ± 39.12	0.002	1.009	1.003−1.01
HDL cholesterol (mg/dl)	37.84 ± 9.47	37.54 ± 9.33	0.77	−	−
Triglycerides (mg/dl)	186.33 ± 101.38	177.19 ± 144.43	0.45	−	−

*Mean ± standard deviation; CAD 5 patients with coronary artery disease; NCA 5 patients with normal coronary arteries.

In the correlation studies, free testosterone showed a weakly negative correlation with CRP (*p* = 0.03, *r* = −0.096) and Lp(a) (*p* = 0.01, *r* = −0.12). Moreover, age had a strong negative correlation with DHEA (*p* < 0.001, *r* = −0.33). However, triglyceride, total cholesterol, LDL and HDL cholesterol levels did not correlate with TES, FREET and DHEA.

**Fig. 1.**
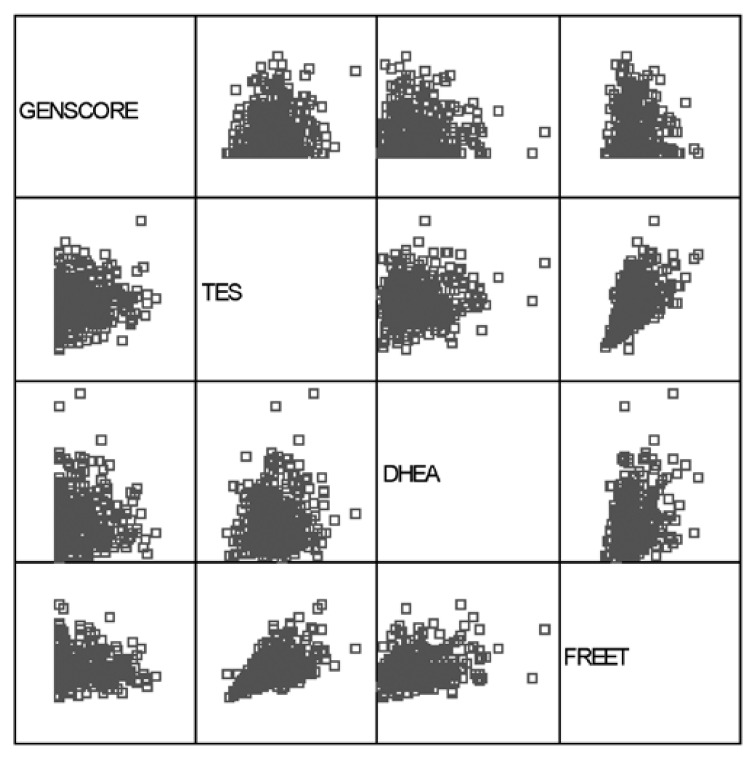
Two-by-two correlations between GENSCORE (Gensini score), TES (total testosterone), DHEA (dehydroepiandrosterone sulfate), and FREET (free testosterone). To read the correlations, extend vertical or horizontal lines from any two variables lying on the main diagonal. The point that these two lines meet represents the scatter plot for these two variables. As an example, the scatter plot that is situated in the bottom corner of the figure represents the correlation between free testosterone and the Gensini score. The more linear, the higher the likelihood of a correlation; the highly scattered plots represent weaker correlations.

**Fig. 2.**
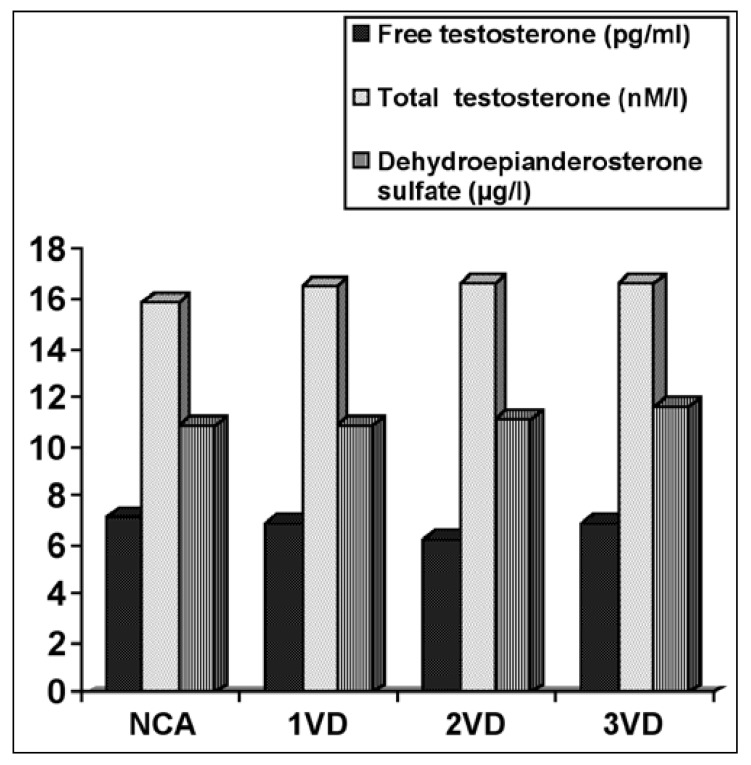
Mean concentrations of free testosterone, total testosterone and dehydroepiandrosterone sulfate in patients with normal coronary arteries and those with one-, two-, and three-vessel disease. NCA = normal coronary arteries, 1VD = one-vessel disease, 2VD = two-vessel disease, 3VD = three-vessel disease.

When we analysed the data in order to find the possible correlation of androgen levels with severity of CAD, no correlation was found between the Gensini score and androgen levels in our study population (Fig. 1, Table 3). Moreover, concentrations of DHEA, FREET and TES did not differ between patients with normal coronary arteries, single-, two-, and three-vessel disease (Fig. 2).

**Table 3:** Correlations Between Levels Of FREET, TES And DHEA With The Gensini Score

*Hormone*	p*-value*	r
FREET	0.30	−0.06
TES	0.88	0.009
DHEA	0.23	−0.07

When we subdivided all subjects into diabetic and non-diabetic groups, in patients with diabetes mellitus, levels of DHEA were significantly lower than in non-diabetics (94.5 ± 59.19 μg/dl vs 117.97 ± 74.54 μg/dl, *p* = 0.004). After subdividing patients with CAD into diabetic and non-diabetic groups, hormone levels did not show any difference between the two groups.

In multivariable logistic regression models, subjects with and without CAD were adjusted for these variables: age, hyperlipidaemia, diabetes mellitus, Lp(a), smoking, total cholesterol and LDL cholesterol. The influence of androgen levels on CAD was assessed after adjustment for these variables. However, the results did not change, and no independent association was found between androgenic hormones and the presence of CAD.

## Discussion

The correlation between androgens and CAD has been debated widely in recent years. Experimental models have shown controversial results on the role of androgens in CAD.^12^,^17^,^18^ These animal models have highlighted the existence of many different mechanisms in the evolution of atherosclerosis that could potentially be influenced by androgens. The inconsistent and conflicting results of these studies may reflect the complexity of pathogenesis of CAD.

As mentioned in the methods section, we excluded 217 patients undergoing selective angiography from our analysis. Most of the exclusions were due to recent MI. In our centre, most patients admitted due to acute MI undergo angiography during their hospitalisation, unless contraindicated. We decided to exclude these patients because CRP, lipid profile and hormone levels might be affected by the acute MI.

On the other hand, 22.7% of our participants had normal coronary arteries or minimal lesions. This figure seems larger than that reported for normal angiograms in other series. However, it is notable that most of those patients did not have completely normal angiograms, but lesions with minimal stenosis (less than 50%), who were classified in this group based on clinical vessel score.

In our study, androgen levels were not significantly different in subjects with and without significant CAD. Some baseline characteristics such as age, diabetes mellitus and BMI may have influenced androgen levels. For example, it has been shown that total and free testosterone and dehydroepiandrosterone sulfate decline with advancing age.^19^,^20^ Also, the association of diabetes mellitus with subnormal free testosterone has been shown in previous studies.^21^ Moreover, it has been shown that hypogonadal men have a higher BMI and obese young men have reduced testosterone levels.^22^

In our study population, BMI was similar in patients with and without CAD. However, because of the difference in age and prevalence of diabetes mellitus between these two groups, we investigated the association of androgenic hormones and CAD in a multivariable logistic regression test (parameters are mentioned in statistical analysis and results sections). Results again did not reveal any association between androgenic hormone levels and CAD.

Several case-control studies have shown either no association^23^–^25^ or a negative association^10^ between androgenic hormone levels and CAD in men. In two prospective studies, it was shown that androgens had no significant relationship with or predictive value for the incidence of CAD.^26^,^27^ Unless no prospective studies had been conducted on this subject, we could have concluded that the results of our study might have been confounded by the general limitations of cross-sectional studies. However, eight great prospective studies have all failed to show any relationship between levels of these hormones and CAD,^12^ which is similar to our results (Tables 1, 2). In addition, a very recent study showed that serum testosterone and DHEA levels were not significantly associated with the incidence of CAD, and only higher serum estradiol levels were associated with lower risk for CAD events in older men.^28^

However, there are other case-control studies showing contradictory results. For example, two studies have shown that men with CAD have significantly lower levels of androgens than normal controls.^29^,^30^ Similarly, in another study, CAD was associated with low serum free testosterone levels.^31^

To our knowledge, the relationship between the severity of CAD and androgen levels has not been discussed in previous studies. The results of a study showed no association between the number of involved vessels and androgen levels.^10^ In addition to the number of involved vessels, we studied the possible association between the Gensini score (which is a more accurate index for severity of CAD) and androgen levels. Finally, we found no association between androgen levels and not only the number of involved vessels, but also with the Gensini score (Table 3, Figs 1, 2).

The other aim of this study was to investigate the correlation of androgens with CRP, total cholesterol, LDL and HDL cholesterol and Lp(a) levels. Plasma androgens have shown positive correlations with HDL cholesterol and negative correlations with total and LDL cholesterol and triglycerides in some studies,^32^–^35^ but, no correlation has been reported between Lp(a) and androgen levels.^36^,^37^ Conversely, we found a weakly negative correlation between androgens and Lp(a), and no correlation between androgen levels and triglyceride, total cholesterol, LDL, and HDL cholesterol levels.

It should be noted that in several studies, after adjustment for BMI, waist circumference, amount of visceral fat, and serum levels of leptin, insulin and free fatty acids (FFA), the correlations of total cholesterol, HDL and LDL cholesterol, and triglyceride levels with testosterone levels lost their statistical significance.^12^ Similarly, in another study, after adjustment for BMI, only the negative correlations of testosterone with insulin and triglycerides remained statistically significant.^38^ Finally, in another study, no correlation was found between serum androgen levels and lipids.^31^

CRP has been found to have a negative correlation with free and total testosterone levels in previous studies.^18^,^19^ In our study, CRP showed a weakly negative correlation with FREET, which may have been due to the small sample size.

The association of diabetes mellitus with subnormal free testosterone levels has been shown in some previous studies.^20^ In our study, however, only DHEA levels were significantly lower in diabetics compared to non-diabetics.

One of the limitations of this study was its cross-sectional nature. Besides, we only assessed the concentrations of free and total testosterone, and did not measure luteinising hormone, oestradiol and serum hormone-binding globulin. The last determinant is important for measurement of bioavailable testosterone, which has been proposed to be a more accurate marker for androgenic activity. On the other hand, when we classified the patients based on clinical vessel score, those with angiographically normal coronary arteries were not separated from patients with minimal non-significant coronary lesions. Therefore, we also assessed the relationship between the Gensini score and hormone levels, by which even minimal coronary lesions were analysed separately from completely normal angiograms.

In conclusion, this study showed that patients with CAD had similar levels of androgenic hormones to those with normal coronary arteries. Moreover, concentrations of these hormones did not vary with different severities of coronary artery disease.
